# The challenges of obesity for fertility: A FIGO literature review

**DOI:** 10.1002/ijgo.14538

**Published:** 2023-01-12

**Authors:** Divya Gautam, Nikhil Purandare, Cynthia V. Maxwell, Mary L. Rosser, Patrick O'Brien, Edgar Mocanu, Ciaran McKeown, Jaideep Malhotra, Fionnuala M. McAuliffe, the FIGO Committee on Impact of Pregnancy on Long‐term Health and the FIGO Committee on Reproductive Medicine, Endocrinology and Infertility

**Affiliations:** ^1^ Department of Obstetrics and Gynecology University Hospital Galway, National University of Ireland Galway Ireland; ^2^ Maternal Fetal Medicine, Sinai Health and Women's College Hospital University of Toronto Toronto Ontario Canada; ^3^ Department of Obstetrics and Gynecology Columbia University Irving Medical Center New York New York USA; ^4^ Institute for Women's Health University College London London UK; ^5^ RCSI Department of Reproductive Medicine Rotunda Hospital Dublin Ireland; ^6^ Malhotra Nursing and Maternity Home Agra India; ^7^ Rainbow Hospital Agra India; ^8^ UCD Perinatal Research Centre, School of Medicine University College Dublin, National Maternity Hospital Dublin Ireland

**Keywords:** adipokines, barriers to fertility treatments, childlessness, infertility, obesity, obesity and social stigma

## Abstract

Obesity has been linked to infertility through several mechanisms, including at a molecular level. Those living with obesity face additional barriers to accessing fertility treatments and achieving a successful pregnancy, which can contribute to their economic and psychosocial stressors. There is scope to further improve care for people living with obesity and infertility with empathy, via a multidisciplinary approach.

## Synopsis

Obesity is linked to infertility at a molecular level and those living with obesity face additional barriers in accessing fertility treatments, which contribute to economic and psychosocial stressors.

## 1 INTRODUCTION

Obesity is a multifactorial disease that adversely affects lives worldwide and is increasingly at the center of modern healthcare cost, policy, and intervention.^1^ Obesity is the product of several determinants that can be divided into upstream causes (policies by the government and economically driven factors), midstream causes (dependent on employment status, level of education), and downstream causes (behavioral and lifestyle).^2^ The agreed World Health Organization (WHO) classification of overweight and obesity for adults is a body mass index (BMI) of greater than or equal to 25 kg/m^2^ and greater than or equal to 30 kg/m^2^, respectively.^3^ Subfertility is defined as the “inability to conceive after 1 year of regular unprotected intercourse” and affects one in seven couples.^4^ The pathophysiology of subfertility is multifactorial. Male and female factor subfertility have been extensively researched to provide targeted investigations and potential solutions for affected couples. Obesity has also been linked to subfertility at a molecular level.^5^

## 2 IMPACT

The latest WHO update from 2017 shows that, since 1975, global obesity rates have nearly tripled.^3^ This encompasses more than 650 million adults aged 18 years and older in 2016. Data also show that over 340 million children and adolescents aged 5–19 years were overweight or obese in 2016.^3^

In the Childhood Determinants of Adult Health (CDAH) study,^6^ a total of 1544 Australian schoolgirls aged 7–15 years in 1985 were followed for 25 years to evaluate whether childhood obesity is associated with infertility in women's reproductive‐aged life. The results demonstrated that childhood obesity before 12 years of age appears to increase the risk of female infertility in later life.^6^

Viewing these WHO data from the perspective of reproductive medicine and evidence from the CDAH study, these 340 million children and adolescents may be among those attending fertility and pregnancy services in the foreseeable future.

## 3 PATHOPHYSIOLOGY

Excess adipose tissue in women increases the risk of subfertility for several reasons. The best understood association between obesity and subfertility is ovulation versus anovulation.^7^ Hormone dysregulation and a combination of insulin‐related disorders, low sex hormone binding proteins, and high levels of androgens play a role. In combination, these cause dysfunction of the hypothalamic gonadotropins hormone secretion, resulting in a reduction in the number of follicles as well as progesterone levels.^7^

White adipose tissue is a secretory and storage organ with multiple functions.^8^ By secreting cytokines, white adipose tissue plays a role in metabolism. One class of cytokines in particular, known as adipokines, has been linked with subfertility.^8^ These include leptin, ghrelin, resistin, visfatin, chemerin, omentin, and adiponectin.^5^ Abnormalities of these, or a disruption in homeostasis, can affect cell function.^8^ For example, high levels of adipose tissue in women exacerbates polycystic ovary syndrome due to increased levels of harmful adipokines and decreased levels of beneficial adipokines.^5^ Receptors for adiponectin are expressed in reproductive tissues, predominantly in the ovaries, endometrium, and placenta. Reduced expression of these receptors has been linked to recurrent implantation failure.^9^ Not only has decreased endometrial receptivity been linked to a decline in adiponectin, but the risk of diabetes increases because one of the roles of adiponectin is uptake of glucose in the liver.^8^ This impairs sensitivity to insulin, linking obesity to insulin resistance and type 2 diabetes mellitus. Chemerin, on the other hand, impairs follicle stimulating hormone (FSH) secretion.

Ovarian follicles are also affected by obesity in other ways. Studies have shown that non‐esterified fatty acid composition in follicular fluid is influenced by the process of lipolysis.^10^ Furthermore, follicular fluid contains high levels of oleic acid in patients with high BMI and this can lead to embryo fragmentation.^9^ When an embryo is in the blastomere stage, chemicals such as stearic acid (also found at elevated levels in follicular fluid of women with high BMI) can lead to poor blastomere score. In mice models, leptin‐rich mice demonstrated impaired folliculogenesis, as well as impaired and reduced rates of ovulation, as leptin inhibits estriol production by these granulosa cells, which are luteinizing hormone (LH) driven. Faster rates of apoptosis in granulosa cells were also seen.^11^ Oocytes of women who are obese or overweight have lower levels of n‐3 polyunsaturated fatty acids compared with oocytes of women with normal weight.^12^ Obesity and presence of adipose tissue have also been linked to increased amounts of inflammation in the body, and not only increase tumor necrosis factor (TNF) but also markers like C‐reactive protein (CRP) and interleukin.^13^

Regarding semen and sperm quality, the pathophysiology of deleterious effects that obesity has is similar to the effect on follicles and oocytes. Oligospermia and azoospermia have been observed in obese males.^14^ Furthermore, the combination of insulin resistance and increased peripheral conversion of androgens to estrogens disrupts hypothalamic signaling. This inevitably affects the circulating level of testosterone, adversely affecting fertility.

## 4 PSYCHOSOCIAL AND ECONOMIC IMPLICATIONS

Subfertility, on its own, carries a risk of mental health issues including depression.^15^ Undertaking fertility investigations can be a massive social and financial undertaking in many societies.^15^

The ability to have children is considered the social norm in many societies and couples affected by infertility may be subject to victimization.^16^ People living with obesity may already face victimization in their daily lives due to their obesity. A study by Puhl and Heuer^17^ described stigma and discrimination toward people living with obesity and reported that attitudes toward this group were often negative and associated with preconceived ideas about laziness, poor hygiene, and unintelligence. Van Balen et al.^18^ undertook a review that demonstrated the range of social and cultural consequences of being childless in resource‐poor settings. These included ridicule and harassment by in‐laws to isolation and impact on inheritance rights. This highlights that, in some societies, having children is considered such an integral part of life that couples that are unable to have children can become outcasts. Furthermore, in countries such as Nigeria, childlessness can affect a person's burial rights, and has even been linked to witchcraft.^18^ A large proportion of resource‐poor countries do not have access to assisted reproductive technology (ART), regardless of BMI, due to its prohibitive cost.^19^

Subfertility, and its accompanying challenges, invariably also impacts a couple's relationship. Most literature focuses on the effect of failed ART on marital relationships. A systematic review of 18 studies of quantitative data demonstrated that lower marital satisfaction was more common in infertile compared with fertile females.^20^ On the contrary, a comparative study with 1528 female participants showed a total of 1402 (78.7%) women with high marital satisfaction and no significant differences in marital communication and conflict resolution between fertile and infertile women.^21^ There are still few studies on the perspectives of infertile males on marital relationships; the few there are, such as the study by Ramezanzadeh et al.,^22^ report that men have decreased sexual satisfaction as a result of their experience with subfertility.^22^

There is a significant economic burden associated with obesity as well as with subfertility; however, it has not been found that the cost of fertility treatments is higher for those with a higher BMI. Increased BMI itself has been associated with a decrease in income. One BMI point above 30 has been associated with a 2% decrease in income, a 3% increase in social transfer payments, and a 4% increase in healthcare costs.^23^ One may wonder if this affects the cost borne for fertility treatments. A retrospective study from 2018 of 826 subfertility patients, grouped by BMI, who accessed intracytoplasmic sperm injection (ICSI) or in vitro fertilization (IVF), demonstrated that the cost of this procedure was not significantly different in women who were overweight or obese^24^ (see section summary in Box 1).

BOX 1 **Section 4 summary**

**Table:** 

**Pathophysiology** White adipose tissue plays a role in metabolism and secretes cytokines; a subtype (adipokines) has been linked with subfertility by affecting cell function High levels of adipose tissue in women exacerbates polycystic ovary syndrome Adiponectin is associated with an increased risk of diabetes and has been linked to recurrent implantation failure The composition in follicular fluid is affected by increased adiposity and can lead to embryo fragmentation, poor blastomere score, and impaired folliculogenesis Oligospermia and azoospermia have been observed in obese males **Psychosocial and economic implications** People living with obesity face stigma and discrimination and can become outcasts in certain societies as childlessness can affect a person's burial rights, inheritance rights, and has been linked to witchcraft There is no clear established link between an increased BMI and higher costs associated with fertility treatments, although women with higher BMIs have a lower chance of live birth per cycle which may invariably affect cost

## 5 ACCESS TO ASSISTED REPRODUCTIVE TECHNOLOGY AND INEQUITIES

The window for embryo implantation is a short period of time when the endometrium is receptive to blastocyst implantation as a result of the action of estrogen and progesterone.^25^ A 2020 review of 68 studies showed that each one unit increase in BMI decreased the probability of implantation after ART by 2.2%–4.3%.^26^ According to Pantasri et al.,^27^ aberrant gene expression in obese women may contribute to a less favorable window, creating a risk of recurrent implantation failure. More recent studies demonstrate this using technologies such as endometrial receptivity analysis (ERA).^28^ A meta‐analysis of 49 studies found a lower live birth rate after ART in overweight and obese women compared with those with normal BMI.^29^ Another meta‐analysis of 115 158 male participants from 30 studies demonstrated that obese men had a higher odds ratio of infertility and a lower odds ratio of live births per cycle; a large proportion of these men had abnormalities in their semen.^30^ A meta‐analysis of 53 studies with 1 445 406 treatment cycles showed that an increased BMI was associated with a weak negative impact on outcomes among women undergoing IVF or ICSI.^31^ This shows that overweight and obesity in this group are associated with an increased number of complications that may prevent a successful pregnancy outcome, thereby increasing cost to the patient and the healthcare system.

Although the evidence in the literature has not clearly demonstrated a significant cost difference, couples living with obesity may not be able to access fertility treatments due to protocols and restrictions by facilities. The UK National Institute for Health and Care Excellence (NICE) guidelines do not explicitly advise against providing IVF to women with obesity, but flag that obesity may impact fertility and recommend providing certain weight loss advice to those women. A report by the National Infertility Group for Scotland, however, does make specific recommendations that women with a BMI over 30 should not be offered fertility treatment.^32^ A 2014 survey of Canadian medical directors found that 50% of respondents imposed a BMI cut‐off for offering fertility treatments.^33^ A study in the USA surveyed 347 clinics that offered IVF services; 120 reported that they employed a weight/BMI cut‐off range using a point system as a determining factor for whether or not the couple/woman was eligible for IVF treatment. Half of these clinics did not offer any weight loss recommendations for treatment.^34^

The Society for Assisted Reproductive Technology (SART) registry contains information from 239 127 IVF cycles using fresh embryos from the years 2008–2010.^35^ Of these, 6000 cycles were in women with a BMI between 30 and 40, and almost 1000 cycles were in women with a BMI over 40. The study showed a decline in the number of oocytes retrieved, as well as the number of embryos of high quality, directly proportional to the rise in BMI. As the degree of obesity increases, implantation and live birth rates decline. Women with a BMI of over 50 had a 21.2% chance of live birth per cycle compared with 31.4% in women with normal BMI.^35^

## 6 CURRENT INTERVENTION AND STRATEGY

Lifestyle modifications have been explored and, in theory, should improve infertility caused by the aforementioned mechanisms by decreasing the amount of adiposity and thereby restoring balance to the hypothalamic axis. This would improve ovulation, endometrial receptivity, issues with insulin resistance, and semen parameters. A small study observing 43 men with obesity during a 14‐week weight loss program showed that, after a 15% loss of weight, semen parameters improved.^36^ Medical and surgical options may potentially lead to a more dramatic loss of weight and improve semen parameters further. Overweight and obese people need to reduce their body weight by 5%–10% with the aim of attaining a BMI in the healthy range (less than 25 kg/m^2^). Not only must this weight be reduced, but also maintained. According to Wing and Hill,^37^ weight loss of at least 10% is successfully maintained for longer than a year, from only lifestyle modification, in 20% of patients.

Prepregnancy counselling should be encouraged at a primary care level and targets of weight loss should be discussed.^38^ A 10% reduction in preconception BMI has been associated with a 10% risk reduction in high‐risk pregnancy‐related issues such as gestational diabetes mellitus, pre‐eclampsia, and adverse birth outcomes like stillbirth.^39^ Since obesity has been linked to an increased risk of neural tube defects and vitamin D deficiency, some recommendations include commencing 150 mg aspirin from 12 weeks of gestation, 5 mg of folic acid daily in the first trimester, and 10 μcg vitamin D daily.^39^, ^40^

Medications are also available for the purposes of weight loss. Orlistat is one such medication and is marketed as a weight loss pill. It is indicated in conjunction with a mildly hypocaloric diet and is a long‐acting inhibitor of gastrointestinal lipases.^41^ The manufacturer recommends use for the treatment of obese patients with a BMI greater or equal to 30 kg/m^2^, or overweight patients (BMI ≥ 28 kg/m^2^) with associated risk factors. Treatment with orlistat should be discontinued after 12 weeks if the patient has been unable to lose at least 5% of their body weight as measured at the start of therapy. A study of 90 participants to assess the efficacy of orlistat compared with metformin and exercise in women with polycystic ovary syndrome (PCOS) showed that orlistat is as effective as metformin and exercise in reducing weight, and achieves similar ovulation rates in obese PCOS patients, with fewer side effects.^42^ Other medications include GLP‐1 analogues, which have been shown to improve the regularity of menstrual cycles in overweight/obese women with PCOS, preconception, thereby increasing the rate of fertility.^43^ GLP‐1 analogues affect fertility parameters in a multifactorial way, by increasing LH surge, reducing inflammation and oxidative damage, and also by affecting the metabolism of lipids and transport of glucose.^44^ In patients with a BMI of 35 or more with medical comorbidities or patients with a BMI over 40, bariatric surgery is recommended,^45^ where available. Not only does the surgery improve a patient's cardiovascular and mortality risk profile, but it can also improve ovulation and fertility among women.^46^ There are still few studies that analyze the impact of bariatric surgery on female and male fertility. Sleeve gastrectomy is a form of laparoscopic bariatric surgery that can bring about longer‐term weight reduction. An abstract presented at the International Federation for the Surgery of Obesity and Metabolic Disorders (IFSO) showed that of 156 female patients who underwent sleeve gastrectomy, 80% showed a reduction of hirsutism, 80% a reduction of radiological evidence of PCOS, and the 132 patients with menstrual irregularities saw a return to normal within 6 months after the operation.^47^ Furthermore, four of the 11 patients who had been subfertile preoperatively and had failed treatments became pregnant postoperatively (see section summary in Box 2).

BOX 2 **Section 6 summary**

**Table:** 

**Access to assisted reproductive technology and inequities** NICE guidelines flag that obesity may impact fertility and recommend providing certain weight loss advice to those women The National Infertility Group for Scotland makes specific recommendations that women with a BMI over 30 should not be offered fertility treatment Respondents to surveys in Canada and the USA reported imposing a BMI cutoff for offering fertility treatments **Current intervention and strategy** There is minimal literature on the direct effect of lifestyle modification on fertility; however, data show a positive effect may be seen if the weight loss is maintained Medications such as orlistat and GLP‐1 analogues have shown promising effects on fertility by improving ovulation rates and regulating the menstrual cycle respectively Bariatric surgery has shown to improve a patient's cardiovascular and mortality risk profile, as well as ovulation and fertility among women

## 7 AREAS FOR FUTURE RESEARCH

- Preconception interventions for women and men living with obesity.
- Direct impact of lifestyle intervention, bariatric surgery, and weight loss on male and female infertility.
- Economic implication of recurrent implantation failure/failed assisted reproductive cycles on fertility centers.
- Perceptions of women/men undergoing fertility investigations and treatment and the effect on their personal relationships and professional life.

## 8 CONCLUSION

The available literature shows that those living with obesity have increased barriers to fertility and accessing treatments available to the general population. There is considerable scope to further improve practice, in that obesity must be addressed as a potentially conquerable barrier to fertility.^11^ This would help decrease psychosocial burden. More research is required on the effects of suggested interventions on the subfertility of people living with obesity to inform policy and setting precedent, globally, on how to manage these concurring issues with empathy and evidence‐based medicine in a multidisciplinary way.

## AUTHOR CONTRIBUTIONS

Divya Gautam and Nikhil Purandare were involved in planning the paper and writing and editing the manuscript. Cynthia Maxwell, Mary Rosser, Patrick O'Brien, Edgar Mocanu, Jaideep Malhotra, Ciaran McKeown, and Fionnuala McAuliffe discussed, commented on, and edited multiple drafts of the paper. Fionnuala McAuliffe approved the final manuscript.

## ACKNOWLEDGMENTS

Open access funding provided by IReL.

## CONFLICT OF INTEREST

Cynthia Maxwell reports grants from the Canadian Institutes for Health Research and the Crohns and Colitis Foundation of Canada. Other authors have no conflicts of interest to declare.
